# Statin Use in COVID-19 Hospitalized Patients and Outcomes: A Retrospective Study

**DOI:** 10.3389/fcvm.2022.820260

**Published:** 2022-02-24

**Authors:** Hamideh Kouhpeikar, Hamidreza Khosaravizade Tabasi, Zahra Khazir, Armin Naghipour, Hussein Mohammadi Moghadam, Hasan Forouzanfar, Mitra Abbasifard, Tatiana V. Kirichenko, Željko Reiner, Maciej Banach, Amirhossein Sahebkar

**Affiliations:** ^1^Department of Hematology and Blood Bank, Tabas School of Nursing, Birjand University of Medical Science, Birjand, Iran; ^2^Department of Nursing, Tabas School of Nursing, Birjand University of Medical Science, Birjand, Iran; ^3^Department of Biostatistics and Epidemiology, Hamadan-Iran Clinical Research Development Center, Imam Reza Hospital, Hamadan University of Medical Sciences, Kermanshah University of Medical Sciences, Kermanshah, Iran; ^4^Immunology of Infectious Diseases Research Center, Research Institute of Basic Medical Sciences, Rafsanjan University of Medical Sciences, Rafsanjan, Iran; ^5^Department of Internal Medicine, Ali-Ibn Abi-Talib Hospital, School of Medicine, Rafsanjan University of Medical Sciences, Rafsanjan, Iran; ^6^Laboratory of Cellular and Molecular Pathology of Cardiovascular System, AP Avtsyn Research Institute of Human Morphology, Moscow, Russia; ^7^Department of Internal Medicine, School of Medicine, University Hospital Centre Zagreb, University of Zagreb, Zagreb, Croatia; ^8^Department of Preventive Cardiology and Lipidology, Chair of Nephrology and Hypertension, Medical University of Lodz, Łódź, Poland; ^9^Cardiovascular Research Centre, University of Zielona Gora, Zielona Gora, Poland; ^10^Applied Biomedical Research Center, Mashhad University of Medical Sciences, Mashhad, Iran; ^11^Biotechnology Research Center, Pharmaceutical Technology Institute, Mashhad University of Medical Sciences, Mashhad, Iran; ^12^School of Medicine, The University of Western Australia, Perth, WA, Australia; ^13^Department of Biotechnology, School of Pharmacy, Mashhad University of Medical Sciences, Mashhad, Iran

**Keywords:** COVID-19, statin, mortality, ICU, inflammation

## Abstract

**Background:**

Coronavirus disease 2019 (COVID-19) might affect everyone, but people with comorbidities such as hypertension and cardiovascular disease (CVD) may often have more severe complications and worse outcomes. Although vaccinations are being performed worldwide, it will take a long time until the entire population of the world is vaccinated. On the other hand, we are witnessing the emergence of new variants of this virus. Therefore, effective therapeutic approaches still need to be considered. Statins are well-known lipid-lowering drugs, but they have also anti-inflammatory and immunomodulatory effects. This study aimed to investigate the effects of statins on the survival of COVID-19 hospitalized patients.

**Methods:**

This retrospective study was performed on 583 patients admitted to a highly referenced hospital in Tabas, Iran, between February 2020 and December 2020. One hundred sixty-two patients were treated with statins and 421 patients were not. Demographic information, clinical signs, and the results of laboratory, and comorbidities were extracted from patients' medical records and mortality and survival rates were assessed in these two groups.

**Results:**

The results of the Cox crude regression model showed that statins reduced mortality in COVID-19 patients (HR = 0.56, 95% CI: 0.32, 0.97; *p* = 0.040), although this reduction was not significant in the adjusted model (HRs=0.51, 95%CI: 0.22, 1.17; *p* = 0.114). Using a composite outcome comprising intubation, ICU admission, and mortality, both crude (HR = 0.43; 95% CI: 0.26, 0.73; *p* = 0.002) and adjusted (HR = 0.57; 95% CI: 0.33, 0.99; *p* = 0.048) models suggested a significant protective effect of statin therapy.

**Conclusion:**

Due to anti-inflammatory properties of statins, these drugs can be effective as an adjunct therapy in the treatment of COVID-19 patients.

## Introduction

The new SARS-CoV-2 corona virus causing COVID-19 disease is responsible for the COVID 19 pandemic. This virus causes, among other harmful effects, an inflammatory condition leading to acute respiratory distress syndrome (ARDS) in patients (1). Everyone is at risk for complications of COVID-19, but people with comorbidities, such as pulmonary diseases, malignant diseases, cardiovascular diseases, hypertension, dyslipidemia, and diabetes are considered as a high-risk group that may have more severe complications. Statins or 3-hydroxy-3-methylglutaryl coenzyme A reductase (HMG-CoA) inhibitors are primarily lipid-lowering drugs. Statins do not only decrease elevated total and LDL-cholesterol in serum and therefore are effective in preventing atherosclerotic cardiovascular disease but also have pleiotropic effects. Their protective effects include anti-inflammatory, antioxidant, antithrombotic, and immune modulatory effects (2–8).

Studies have reported that statins may inhibit COVID-19 infection by binding to a key virus protease (Mpro). This protease plays an important role in proteolytic maturation (9). Coronaviruses also stimulate an inflammatory cascade by interaction with Toll-like receptors on the membrane of host cells and activating of NF-κB pathways. Therefore, inhibition of NF-κB can reduce SARS-CoV-2 infectivity. On the other hand, it has been demonstrated that statins significantly suppressed the activation of NF-κB and exert anti-inflammatory actions (10–12). Observational studies have reported that treatment with statins was associated with a reduced mortality rate in hospitalized patients with influenza (13). Previous studies have shown that statins may be also useful in the treatment of COVID-19 due to their pleotropic effect (14–18).

In addition, since atherosclerotic cardiovascular disease and dyslipidemia are associated with poor prognosis in COVID-19 patients, statins may be also useful in improving the treatment of these patients by reducing the complications of the disease (19). Several mechanisms have been suggested for the efficacy of statins in COVID-19. CD 147, a receptor for SARS-CoV-2 on the membrane of host cells, is a novel route for SARS-CoV-2 invasion and it has been shown that statins could reduce the infectivity of SARS-CoV-2 by reducing the expression of this receptor (20, 21). Although almost all countries have started with vaccination against SARS-CoV-2, due to the emergence of mutated variants of this virus and differences in vaccination rates in different countries, it is not clear when this pandemic will stop. Therefore, looking for more effective therapies for COVID-19 patients will remain an important strategy. Acute respiratory syndrome and thromboembolism are two well-known complications that cause mortality in most of the hospitalized COVID-19 patients (22). Evidence suggests that these complications of COVID-19 are associated with an inflammatory response (23). Recent studies demonstrated that statins reduced the mortality rate by 42% in hospitalized patients with COVID-19 (24). Statins also decreased the risk of acute respiratory distress syndrome (ARDS) (25). On the other hand, some studies have reported that statins may increase the expression of ACE2 (angiotensin converting enzyme 2) and ACE2 is a receptor for SARS-CoV-2 enabling the virus to enter the host cells (26, 27). Therefore, inhibiting the renin-angiotensin system cascade, which is a target of ACE2, might also have some effects in patients with COVID-19 who have cardiovascular disease (28). Anyhow, according to different studies, there are still controversies about the effectiveness of statins in COVID-19 patients. Therefore, the aim of this retrospective study was to investigate the relationship between treatment with statins and the survival rate in hospitalized COVUD-19 patients.

## Materials and Methods

### Study Design

A retrospective study was performed on COVID-19 patients admitted to Shahid Mostafa Khomeini Hospital in Tabas, Iran. Participants were admitted between February 2020 and December 2020. A total of 583 COVID-19 patients with symptoms of the disease and positive polymerase chain reaction (PCR) were included in the study. One hundred sixty-two patients were treated with statins and 421 were in the non-statin group.

Patients in the statin group were all treated with atorvastatin 40 mg daily.

Demographic characteristics, patients' clinical signs and symptoms, comorbidities, laboratory test results, chest computed tomography (CT) scan reports, vital signs, and clinical outcomes were collected from medical patients' records. Laboratory test results included complete blood count (CBC), liver enzymes, urea, creatinine, CRP, and vital signs such as body temperature, spO2, and blood oxygen level. The data on oxygen therapy, mechanical ventilation, and length of hospital stay were collected from medical records. All data were checked by two separate researchers to verify their accuracy.

The endpoints for evaluating the patients' status included the need for oxygen therapy, hospitalization in the intensive care unit (ICU), blood oxygen level, duration of hospitalization, and mortality rate. A secondary composite outcome comprising mortality, ICU admission, and intubation was also considered. Exclusion criteria were: people over 85 years and under 19 years and people with these diseases: hepatitis, AIDS, influenza, and tuberculosis.

This study was approved by the ethics committee of Birjand University of Medical Sciences (IR.BUMS.REC.1400.073).

### Statistical Analysis

Data were analyzed using stata software version 14. In descriptive statistics, data are presented as mean, standard deviation (SD), frequency, and frequency percentage. In analytical statistics, the Kolmogorov-Smirnov test was performed for the normality of continuous variables and the Schoenfeld residual test was performed to check the proportional hazards (pH) assumption in the simple model and Cox multiple model. To determine difference between the mean of HB variable data between the two groups (statin vs. non-statin), an independent *t*-test was used for continuous variables such as PO2 status, body temperature, WBC, platelets, neutrophils and lymphocytes. For non-parametric variables? comparison between groups was performed using Mann-Whitney U test. Fisher's exact test and Chi-square test were used for categorical variables.

Kaplan-Meyer curve was drawn to show the survival time of patients in the two groups—-treated with statins and those not treated with statins. Log-Rank test was performed to check the difference in survival time between the two groups of patients. Cox simple regression model was used to determine the factors related to survival time in patients with COVID-19. These analyses were also carried out to test the effect of statin use on the composite outcome. Multivariable Cox regression model was used for variables that were statically significant in the simple Cox regression model. Hazard ratio (HR) and 95% confidence interval (CI) were reported for each of the variables related to patient survival time in two simple models and Cox multivariable model. The significance level was 0.05.

## Results

The total number of COVID-19 patients who were included in this study was 583. One hundred sixty-two patients were treated with atorvastatin (statin group) and the remaining 421 were not treated with statin (non-statin group). The mean age of the patients was 60 years and 52.3% of participants were male. There was no significant relationship between patients' age and statin use. Symptoms of hospitalized patients are listed in Table 1. The most common symptoms included fever, cough, respiratory distress, nausea, and muscle aches. Comorbidities of these patients are listed in Table 2. The most common comorbidity was hypertension (26%), followed by cardiovascular diseases (including unstable angina, stable angina, myocardial infarction, congestive heart failure and coronary heart disease) (25%). The prevalence of hypertension was higher in the statin group (33.9 vs. 23.9% in non-statin group). Cardiovascular diseases were more often in non-statin group (27.3 vs. 19.1% in the statin group). 50% of patients had pO2 <93%, which was similar in both groups. The need for oxygen therapy in the statin group was higher than in the non-statin group (30 vs. 15%). The mean time between onset of symptoms and hospitalization of patients was 4.7 days, which was similar in both groups.

**Table 1:** Characteristics of patients in statin and non-statin groups.

**Variable**	**Level variable**	**Total (*n* = 582)**	**No statin (*n* = 421, 72.2%)**	**Atorvastatin (*n* = 162, 27.8%)**	**Test result *p*-value**
Age (mean ± S.D)		61.4 ± 0.9	61.6 ± 21.4	60.8 ± 17.9	M.W = 32849.5 *p =* 0.492
Sex	Female (n, %)	278 (47.7)	189 (68.0)	89 (32.0)	χ^2^ = 4.73
	Male (n, %)	305 (52.3)	232 (76.1)	73 (23.9)	*p =* 0.030
ICU	Non ICU	384 (83.3)	250 (65.1)	134 (34.9)	χ^2^ = 0.06
	ICU	77 (16.7)	49 (63.6)	28 (36.4)	*p =* 0.806
Death	Yes (n, %)	73 (12.5)	56 (76.7)	17 (23.3)	χ^2^ = 24.34 *p =* 0.354
Hospitalization time (mean ± S.D)		5.5 ± 9.3	5.5 ± 10.6	5.8 ± 4.0	M.W = 32849.5 ***p** **=*** **0.001**
**Sign and symptoms**					
Fever	Yes (n, %)	289 (49.6)	206 (71.3)	83 (28.7)	χ^2^ = 0.25 *p =* 0.618
Cough	Yes (n, %)	212 (36.4)	142 (67.0)	70 (33.0)	χ^2^ = 4.54 ***p** **=*** **0.033**
Muscular pain	Yes (n, %)	66 (11.3)	40 (60.6)	26 (39.4)	χ^2^ = 4.99 ***p** **=*** **0.025**
ARDS	Yes (n, %)	203 (34.8)	135 (66.5)	68 (33.5)	χ^2^ = 5.06 ***p** **=*** **0.024**
Consciousness	Yes (n, %)	33 (5.7)	25 (75.8)	8 (24.2)	χ^2^ = 0.22 *p =* 0.640
Olfactory problems	Yes (n, %)	4 (0.7)	4 (100.0)	00 (00.0)	Exact = 1.55 *p =* 0.580
Taste problems	Yes (n, %)	4 (0.7)	4 (100.0)	00 (00.0)	Exact = 1.55 *p =* 0.580
Convulsions	Yes (n, %)	3 (0.5)	3 (100.0)	00 (00.0)	Exact = 1.16 *p =* 0.564
Stomachache	Yes (n, %)	20 (3.4)	18 (90.0)	2 (10.0)	Exact = 3.31 *p =* 0.069
Nausea	Yes (n, %)	81 (13.9)	67 (82.7)	14 (17.3)	χ^2^ = 5.17 ***p** **=*** **0.023**
Vomiting	Yes (n, %)	55 (9.4)	157 (29.7)	5 (9.1)	χ^2^ = 10.58 ***p** **=*** **0.001**
Diarrhea	Yes (n, %)	50 (8.6)	44 (88.0)	6 (12.0)	χ^2^ = 6.79 *p =* 0.009
Anorexia	Yes (n, %)	50 (8.6)	25 (50.0)	25 (50.0)	χ^2^ = 13.45 *p =* 0.001
Headache	Yes (n, %)	38 (6.5)	21 (55.3)	17 (44.7)	χ^2^ = 5.82 *p =* 0.016
Vertigo	Yes (n, %)	17 (2.9)	14 (82.4)	3 (17.6)	Exact = 0.89 *p =* 0.422
Paralysis	Yes (n, %)	1 (0.1)	1 (100.0)	00 (00.0)	Exact = 0.39 *p =* 0.999
Plegia	Yes (n, %)	2 (0.3)	1 (50.0)	1 (50.0)	Exact = 0.49 *p =* 0.479
Chest pain	Yes (n, %)	29 (5.0)	25 (86.2)	4 (13.8)	Exact = 2.98 *p =* 0.092
**Comorbidities**					
Cancer	Yes (n, %)	7 (1.2)	6 (85.7)	1 (14.3)	Exact = 0.64 *p =* 0.680
Liver disease	Yes (n, %)	3 (0.5)	1 (33.3)	2 (66.7)	Exact = 2.27 *p =* 0.188
Diabetes	Yes (n, %)	81 (13.9)	58 (71.6)	23 (28.4)	χ^2^ = 0.02 *p =* 0.895
Hematologic disorders	Yes (n, %)	2 (0.3)	1 (50.0)	1 (50.0)	Exact = 0.49 *p =* 0.479
Immunodeficiency	Yes (n, %)	1 (0.2)	1 (100.0)	00 (00.0)	Exact = 0.39 *p =* 0.999
Cardiovascular diseases	Yes (n, %)	146 (25.0)	115 (78.8)	31 (21.2)	χ^2^ = 4.17 *p =* 0.043
Kidney diseases	Yes (n, %)	12 (2.1)	10 (83.3)	2 (16.7)	Exact = 0.76 *p =* 0.525
Asthma	Yes (n, %)	16 (2.7)	14 (87.5)	2 (12.5)	Exact = 1.93 *p =* 0.257
Neurologic diseases	Yes (n, %)	9 (1.5)	7 (77.8)	2 (22.2)	Exact = 0.14 *p =* 0.999
Hypertension	Yes (n, %)	155 (26.6)	101 (65.2)	54 (34.8)	χ^2^ = 5.23 *p =* 0.022
Oxygen therapy status	Yes (n, %)	117 (36.5)	67 (57.3)	50 (42.7)	χ^2^ = 4.40 *p =* 0.036
**Other**					
The time from the onset of symptoms to hospitalization	mean ± S.D	4.7 ± 2.3	4.6 ± 2.0	4.7 ± 3.0	M.W = 31535.5 *P =* 0.155
Mechanical ventilation	Yes (n, %)	24 (7.5)	16 (10.1)	8 (4.9)	χ^2^ = 3.05 *p =* 0.092
pO2	<93%	286 (50.6)	204 (71.3)	82 (28.7)	χ^2^ = 3.09
	>93%	279 (49.4)	217 (77.8)	62 (22.2)	*p =* 0.079
Number of breaths	14–18	35 (18.9)	4 (11.4)	31 (88.6)	Exact = 0.89
	18–22	122 (65.9)	14 (11.5)	108 (88.5)	*P =* 0.602
	>22	28 (15.2)	5 (17.9)	23 (82.1)	
CT scan	Yes (n, %)	160 (49.8)	47 (29.4)	113 (70.6)	χ^2^ = 51.8 ***p** **=*** **0.001**
Smoking	Yes (n, %)	6 (1.0)	5 (83.3)	1 (16.7)	Exact = 0.37 *P =* 0.541
Opium use	Yes (n, %)	41 (7.0)	34 (82.9)	7 (17.1)	χ^2^ = 2.52 *p =* 0.112
S PO2	mean ± S.D	91.4 ± 6.4	91.6 ± 6.8	90.9 ± 3.5	M.W = 28829.5 *P =* 0.004
Temperature	mean ± S.D	37.0 ± 0.8	37.0 ± 0.9	37.1 ± 0.7	M.W = 19886.5 *P =* 0.003
**Laboratory measures**					
WBC	mean ± S.D	8.5 ± 6.5	9.2 ± 7.2	6.6 ± 3.5	M.W = 21107.5 ***P** **=*** **0.001**
HB	mean ± S.D	12.5 ± 1.8	12.6 ± 1.9	12.4 ± 1.7	*T*.test = *P =*
PLT	mean ± S.D	199.4 ± 93.5	200.2 ± 94.2	197.1 ± 92.0	M.W = 33196.0 *P =* 0.619
Neut	mean ± S.D	74.2 ± 13.8	75.4 ± 13.5	71.3 ± 14.3	M.W = 27989.5 ***P** **=*** **0.001**
Lymph	mean ± S.D	18.8 ± 11.5	18.0 ± 11.6	21.0 ± 10.9	M.W = 27941.0 ***P** **=*** **0.001**
RDW	mean ± S.D	14.1 ± 1.9	14.2 ± 1.9	13.9 ± 1.7	M.W = 30680.5 *P =* 0.060
MPV	mean ± S.D	9.9 ± 1.0	9.9 ± 1.1	9.9 ± 1.1	M.W=30764.5 *P =* 0.067
BUN	mean ± S.D	17.4 ± 12.3	17.6 ± 13.1	16.7 ± 9.9	M.W = 33235.0 *P =* 0.634
Cr	mean ± S.D	1.2 ± 0.9	1.2 ± 0.7	1.3 ± 1.4	M.W = 30424.5 *P =* 0.042
CRP	mean ± S.D	1.1 ± 0.7	1.2 ± 0.8	0.9 ± 0.3	M.W = 25494.0 ***P** **=*** **0.001**

*P-values were calculated by T-test for normally distributed continuous variables or Mann-Whitney U (M.W) test for non-normally distributed continuous variables and Fisher's exact (exact) test or χ^2^ test for the categorical variables; P < 0.05 was considered significant*.

**Table 2:** Data analysis using the crude and adjusted Cox regression models considering mortality as a single outcome.

**Variable**	**Level variable**	**Death (*n* = 73, 12.5%)**	**Crude model**		**Adjusted model**	
			**HR (95% CI)**	***p***	**HR (95% CI)**	***p***
Age (mean ± S.D)		75.8 ± 14.9	1.04 (1.02, 1.06)	0.001	1.02 (1.01, 1.05)	0.034
Sex	Female (n, %)	29(10.5)	Baseline	0.095	–	–
	Male (n, %)	44 (14.4)	1.49 (0.93, 2.38)		–	
ICU	ICU	40 (51.9)	Baseline	0.001	Baseline	0.117
			3.69 (2.18, 6.25)		1.89 (0.85, 4.20)	
Atorvastatin	Yes (n, %)	17 (10.4)	Baseline	0.040	Baseline	0.114
			0.56 (0.32, 0.97)		0.51 (0.22, 1.17)	
**Sign and symptoms**						
Fever	Yes (n, %)	25 (8.6)	Baseline	0.001	Baseline	0.467
			0.44 (0.27, 0.72)		1.30 (0.64, 2.63)	
Cough	Yes (n, %)	22 (10.3)	Baseline	0.027	Baseline	0.711
			0.56 (0.34, 0.93)		0.87 (0.43, 1.76)	
ARDS	Yes (n, %)	34 (16.7)	Baseline	0.367	–	–
			1.23 (0.77, 1.96)		–	
Consciousness	Yes (n, %)	10 (30.3)	Baseline	0.103	–	–
			1.75 (0.89, 3.43)		–	
**Comorbidities**						
Diabetes	Yes (n, %)	15 (18.5)	Baseline	0.133	–	–
			1.54 (0.87, 2.73)		–	
Cardiovascular diseases	Yes (n, %)	26 (17.8)	Baseline	0.011	Baseline	0y.023
			1.86 (1.15, 3.01)		2.37 (1.13, 4.99)	
Asthma	Yes (n, %)	12 (22.6)	Baseline	0.109	–	–
			1.66 (0.89, 3.09)		–	
Hypertension	Yes (n, %)	22 (14.1)	Baseline	0.793	–	–
			1.06 (0.54, 1.76)		–	
Oxygen therapy status	Yes (n, %)	35 (29.9)	Baseline	0.001	Baseline	0.776
			2.89 (1.59, 5.24)		1.11 (0.53, 2.32)	
**Other**						
Intubation	Yes (n, %)	22 (91.6)	Baseline	0.001	Baseline	0.056
			5.25 (2.95, 9.34)		2.16 (0.98, 4.80)	
pO2	<93%	55 (19.2)	Baseline	0.002	Baseline	0.255
	>93%	16 (5.7)	0.41 (0.23, 0.73)		0.58 (0.22, 1.49)	
Number of breaths	14–18	4 (11.4)	Baseline	0.434	–	–
	18–22	13 (10.6)	0.63 (0.20, 1.97)		–	
	>22	12 (42.8)	1.92 (0.60, 6.10)	0.266	–	–
CT scan	Yes (n, %)	22 (13.7)	Baseline	0.874	–	- –
			0.95 (0.54, 1.68)		–	
Opium use	Yes (n, %)	8 (19.5)	Baseline	0.080	–	–
			1.93 (0.92, 4.04)		–	
SPO2 (mean ± S.D)	85.4 ± 10.2		0.94 (0.92, 0.96)	0.001	0.96 (0.92, 0.99)	0.035
Body temperature (mean ± S.D)	36.8 ± 0.6		0.69 (0.47, 1.01)	0.060	–	–
The time from the onset of symptoms to hospitalization (mean ± S.D)	4.4 ± 2.3		0.94 (0.84, 1.04)	0.253	–	–
**Laboratory measures**						
WBC (mean ± S.D)	9.8 ± 4.3		1.02 (1.01, 1.04)	0.012	1.04 (0.93, 1.16)	0.459
HB (mean ± S.D)	12.2 ± 1.8		0.90 (0.79, 1.03)	0.145	–	–
PLT (mean ± S.D)	194.3 ± 87.9		0.99 (0.99, 1.00)	0.399	–	–
Neut (mean ± S.D)	78.0 ± 14.9		1.01 (0.99, 1.03)	0.191	–	–
Lymph (mean ± S.D)	15.3 ± 11.8		0.97 (0.95, 1.00)	0.056	–	–
RDW (mean ± S.D)	15.0 ± 2.2		1.17 (1.06, 1.29)	0.001	0.91 (0.76, 1.09)	0.318
MPV (mean ± S.D)	10.0 ± 1.0		0.99 (0.79, 1.24)	0.941	–	–
BUN (mean ± S.D)	23.8 ± 16.7		1.02 (1.01, 1.04)	0.001	1.01 (0.92, 1.04)	0.198
Cr (mean ± S.D)	1.5 ± 1.8		1.13 (1.01, 1.26)	0.025	1.18 (1.01, 1.39)	0.044
CRP (mean ± S.D)	1.1 ± 0.6		1.03 (0.75, 1.41)	0.835	–	–

*P < 0.05 was considered significant*.

Seventy-seven patients (16.7%) were admitted to the ICU ward. 28 of these patients were treated with statins while 49 patients were in non-statin group. The rate of admission to ICU did not differ significantly between the two groups (*p* = 0.8), A total of 509 (87.5%) patients were discharged from the hospital, and 73 (12.5%) died. 17 (10.4%) patients who died were in the statin group and 56 (13.3%) were in the non-statin group.

The rate of mechanical ventilation was in all patients 7.5%, but it was higher in the group that was treated with statins (4.9 vs. 3.8%). In 49.8% of patients, CT scan was abnormal and showed serious signs of lung involvement which was higher than in patients treated with statins. WBC count was higher in the group that did not take statins than in the statin group (*p* = 0.001). Statin therapy also reduced neutrophiles count (*p* = 0.001).

**Figure 1:**
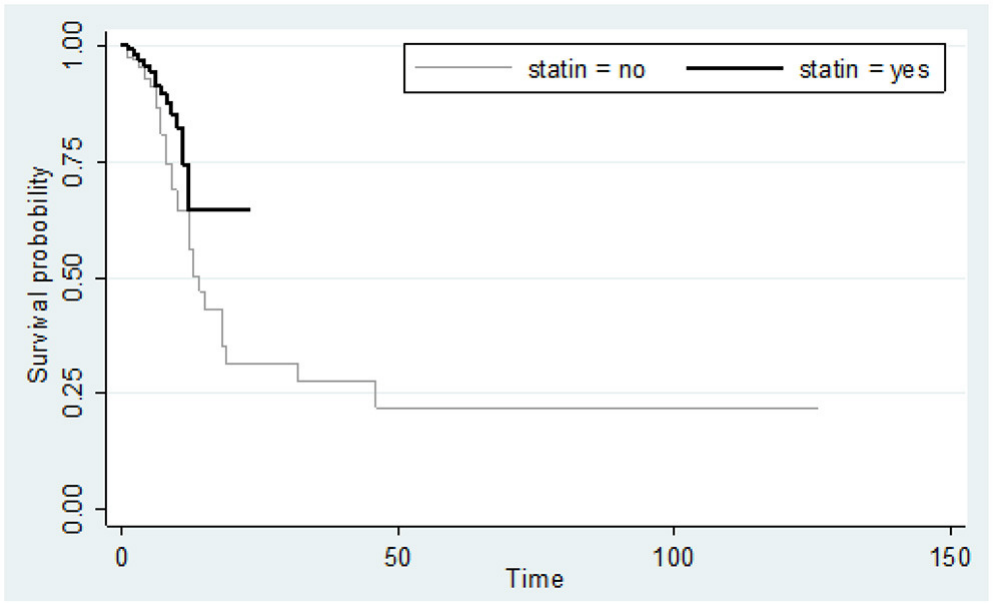
Kaplan-Meier curve comparing the survival time (in days) between statin users and statin non-users considering mortality as an event. Value test log-rank = 4.46; *p*-value = 0.034.

The Kaplan-Meyer curve showed that people who were treated with statins had a longer survival time than those who were not treated. Log-rank statistical test indicated that the survival of patients between the two groups (statin and non-statins) was statistically significant (Figure 1). 14.4 %(*n* = 44) of all deaths were in men. The risk ratio in men was 1.49 higher than women, i.e., survival was lower in men than in women, although the risk ratio in women and men was not significantly different (95% CI = 0.93, 2.38).

Results of Cox regression were reported as crude and adjusted hazard ratios (HRs) and are presented in Table 2. They indicated that with 1-year increase in age? the risk of death in COVID-19 patients increased by 1.02 folds (HR = 1.02, *p* = 0.034). Findings in the crude model indicated that the risk of mortality in the statin group was lower than patients who were not treated with statins, in other words, treatment with statin increased survival time (HR = 0.56, 95% CI = (0.32,0.97) *p* = 0.040). However, the results of Cox multivariable model showed that the reduction of mortality rate in those treated with statin was numerically by 49% but not statistically significant [HRs = 0.51, 95% CI = (0.22, 1.17) *p* = 0.114]. Mortality rate in patients with COVID-19 who had a history of heart disease was 2.37 times higher than in patients without a history of heart disease [HR = 2.37, 95% CI = (1.13,4.99) *p* = 0.023].

Patients who were admitted to ICU and patients who were mechanically ventilated had a higher risk of death than the others, although this difference was not statistically significant.

**Figure 2:**
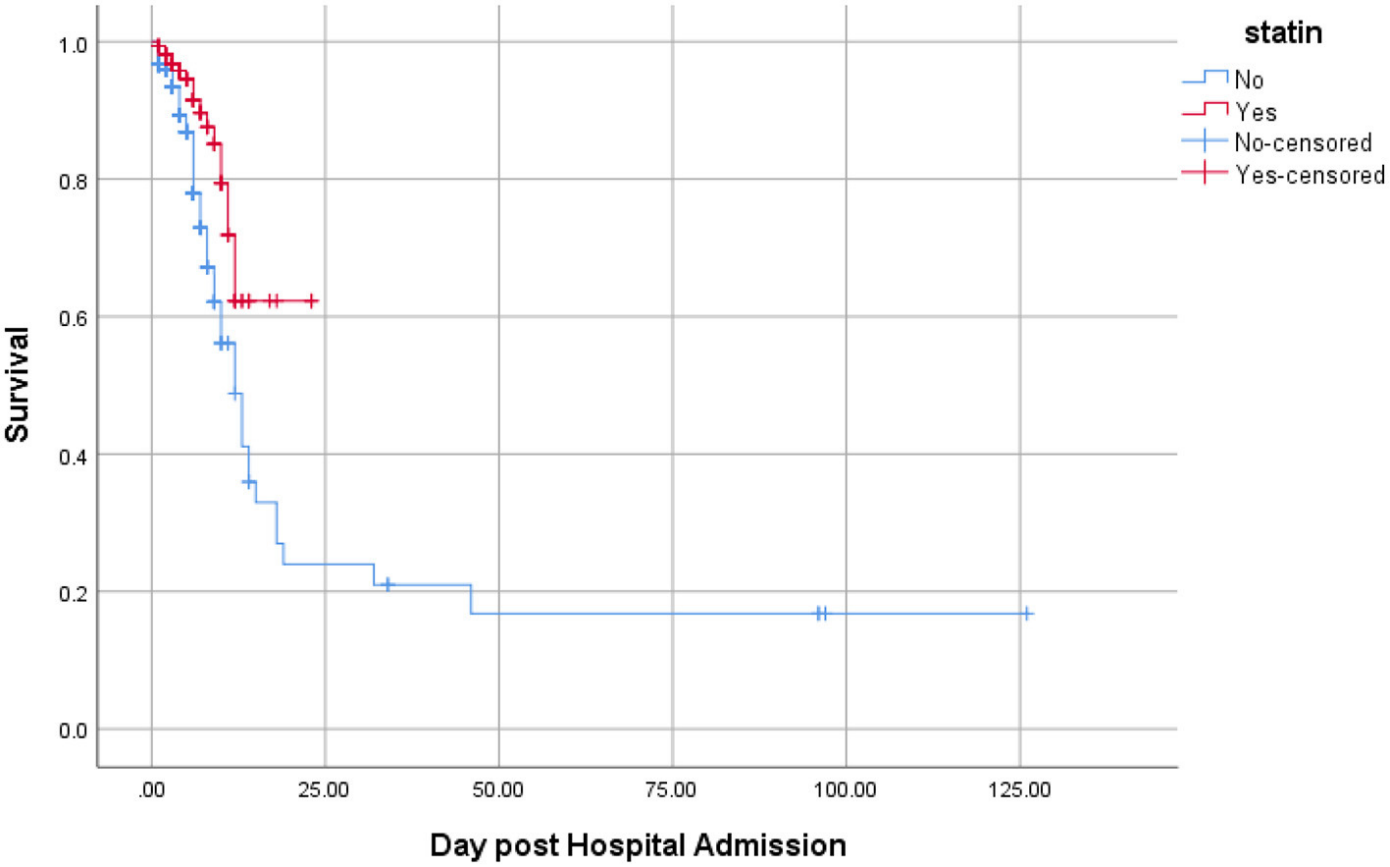
Kaplan-Meier curve comparing the survival time (in days) between statin users and statin nonusers considering the composite of mortality, ICU admission, and intubation as the event. Value test log-rank = 11.06; *p*-value = 0.001.

A secondary analysis was also performed considering a composite outcome comprising intubation, ICU admission, and death. The results of Kaplan-Meyer curve showed that statin use was associated with less composite outcome (Figure 2). Results of Cox regression model are presented in Table 3. The crude model of Cox regression showed patients who used statins were less likely to experience the composite outcome compared with non-statin users [18/162 (11.1%) vs. 77/421 (18.3%); HR = 0.43, 95% CI: 0.26, 0.73; *p* = 0.002). The results of the adjusted Cox multivariable model also confirmed the lower occurrence of composite outcome in statin users vs. non-statin users (HR = 0.57, 95% CI: 0.33, 0.99; *p* = 0.048). Age (HR = 1.02, 95% CI: 1.01, 1.03; *p* = 0.016) and the male gender (HR = 1.82, 95%CI: 1.18, 2.83; *p* = 0.007) were associated with higher rates of composite outcome.

**Table 3:** Data analysis using the crude and multiple Cox regression models for the composite outcome comprising mortality, ICU admission and intubation.

**Variable**	**Level**	**Total (*n* = 583)**	**Event (*n* = 95, 16.3%)**	**Crude model**		**Multiple model**	
				**HR (95% CI)**	***P***	**HR (95% CI)**	***P***
Age (mean ± S.D)		61.4 ± 20.4	73.6 ± 15.5	1.03 (1.02, 1.04)	0.001	1.02 (1.01, 1.03)	0.016
Sex	Female (n,%)	278 (47.7)	35 (12.6)	1 (Baseline)	0.015	1 (Baseline)	0.007
	Male (n, %)	305 (52.3)	60 (19.7)	1.68 (1.11, 2.55)		1.82 (1.18, 2.83)	
Statin	Yes (n, %)	162 (27.8)	8 (11.1)	1 (Baseline)	0.002	1 (Baseline)	0.048
				0.43 (0.26, 0.73)		0.57 (0.33, 0.99)	
**Sign and symptoms**							
Fever	Yes (n, %)	342 (58.7)	62 (18.1)	1 (Baseline)	0.001	1 (Baseline)	0.052
				0.49 (0.32, 0.75)		0.62(0.38, 1.01)	
Cough	Yes (n, %)	417 (71.5)	74 (17.7)	1 (Baseline)	0.012	1 (Baseline)	0.905
				0.53 (0.33, 0.87)		0.91(0.51, 1.59)	
Muscular pain	Yes (n, %)	134 (23.0)	24 (17.9)	1 (Baseline)	0.354	–	–
				1.25 (0.78, 1.98)		–	–
ARDS	Yes (n, %)	139 (23.8)	34 (24.5)	1 (Baseline)		–	–
				1.03 (0.99, 4.28)	0.882	–	–
consciousness	Yes (n, %)	15 (2.6)	8 (53.3)	1 (Baseline)	0.052	–	–
				2.06 (0.99, 4.8)		–	–
Nausea	Yes (n, %)	69 (11.8)	10 (14.5)	1 (Baseline)	0.447	–	–
				1.29 (0.67, 2.50)		–	–
Anorexia	Yes (n, %)	45 (7.7)	5 (11.1)	1 (Baseline)	0.423	–	–
				0.69 (0.28, 1.71)		–	–
**Comorbidities**							
Diabetes	Yes (n, %)	81 (13.9)	15 (18.5)	1 (Baseline)	0.702	–	–
				1.11 (0.64, 1.94)		–	–
Cardiovascular disease	Yes (n, %)	146 (25.0)	32 (21.9)	1 (Baseline)	0.015	1 (Baseline)	0.972
				1.70 (1.11, 2.60)		1.01 (0.62, 1.64)	
Asthma	Yes (n, %)	16 (2.7)	4 (25.0)	1 (Baseline)	0.093	–	–
				2.37 (0.87, 6.50)		–	–
Norologic	Yes (n, %)	9 (1.5)	3 (33.3)	1 (Baseline)	0.960	–	–
				1.03 (0.32, 3.29)		–	–
Hypertention	Yes (n, %)	27 (17.4)	155 (26.6)	1 (Baseline)	0.964	–	–
				0.99 (0.63, 1.55)		–	–
**Other**							
pO2	< =90%	394 (67.8)	83 (21.1)	1 (Baseline)	0.002	1 (Baseline)	0.084
	>90%	187 (32.2)	12 (6.4)	0.30 (0.17, 0.56)		0.56 (0.29, 1.08)	
CT scan	Yes (n, %)	165 (28.3)	25 (15.2)	1 (Baseline)	0.298	–	–
				0.78 (0.49, 1.24)		–	–
smoke	Yes (n, %)	33 (5.7)	8 (24.2)	1 (Baseline)	0.030	1 (Baseline)	0.312
				2.24 (1.08, 4.66)		1.49 (0.69, 3.21)	
Opium	Yes (n, %)	14 (2.4)	7 (50.0)	1 (Baseline)	0.223	–	–
				1.63 (0.74, 3.54)		–	–
temperature	mean ± S.D	37.1 ± 0.7	37.2 ± 0.6	0.98 (0.58, 1.66)	0.951	–	–
**Laboratory measures**							
WBC	mean ± S.D	8.5 ± 6.5	10.1 ± 4.6	1.03 (1.01, 1.04)	0.001	1.02 (0.99, 1.05)	0.271
HB	mean ± S.D	12.5 ± 1.8	12.3 ± 1.9	0.92 (0.81, 1.03)	0.915	–	–
PLT	mean ± S.D	199.4 ± 93.5	199.6 ± 88.7	1.00 (0.99, 1.01)	0.712	–	–
Neut	mean ± S.D	74.2 ± 13.8	79.1 ± 14.4	1.02 (1.01, 1.04)	0.020	1.02 (0.98, 1.06)	0.327
Lymph	mean ± S.D	18.8 ± 11.5	14.7 ± 11.3	0.97 (0.95, 0.99)	0.005	1.02 (0.97, 1.06)	0.491
BUN	mean ± S.D	17.4 ± 12.3	22.9 ± 17.9	1.03 (1.02, 1.04)	0.001	1.04 (1.02, 1.06)	0.012
MPV	mean ± S.D	9.9 ± 1.03	10.0 ± 1.1	0.99 (0.81, 1.20)	0.913	–	–
Cr	mean ± S.D	1.2 ± 0.9	1.5 ± 1.7	1.13 (1.02, 1.25)	0.016	1.18 (0.98, 1.42)	0.079
CRP	mean ± S.D	1.1 ± 0.7	1.2 ± 0.7	1.18 (0.89, 1.54)	0.243	–	–
RDW	mean ± S.D	14.1 ± 1.8	14.9 ± 2.1	1.14 (1.05, 1.25)	0.003	1.06 (0.96, 1.18)	0.235

*Extended Cox Model; P < 0.05 was considered significant*.

## Discussion

In this retrospective study performed on 583 hospitalized COVID-19 patients, the effects of atorvastatin on mortality, ICU admission, and intubation were investigated. Results of the crude regression model showed that statins reduced mortality. However, this reduction in mortality was not significant in the adjusted model, which might be attributed to the low number of events. When the composite outcome was considered, the results of both crude and adjusted models supported a significant protective effect of statin use. Kaplan-Meyer curve also showed that statin use increased survival of hospitalized patients. A recent study in Shiraz also found that statins reduced mortality in hospitalized COVID-19 patients, although this was not statistically significant (29). In a retrospective study on 13,981 COVID-19 patients in Hubei Province, China, 1,219 patients were treated with statins. In our study, it was found that the survival rate in men was lower than women; in other words, the risk ratio in men was 1.49 times higher than women. This is consistent with the excess mortality observed in men in other populations (30). However, the results of our analyses on the impact of statin therapy were adjusted for potential confounders, including gender.

The present results indicated that statins reduced mortality in these patients. The mortality rate in the statin group was 5.2%, while in the non-statin group, this rate was higher (9.4%) (24). In accordance with these data, in a study performed by Masana et al. on 2,157 patients with COVID-19, patients who were treated with statins (*n* = 581) had a lower mortality rate than those who were not treated (31). In contrary to these studies, a cohort study in South Korea on 7,780 COVID-19 patients demonstrated that there was no difference in mortality rate between patients treated with statins and those who were not treated (32). In a retrospective cohort study on 2,191 hospitalized COVID-19 patients, statins were also not independently associated with all-cause mortality during follow-up (33).

Another recent retrospective analysis of 4,447 COVID-19 patients admitted to the hospital found that statin use was not associated with altered mortality and found an close to 20% increased risk of COVID-19 infection (34). It is important to mention the results of some studies in which individuals were treated with statins prior to hospitalization for COVID-19 and had therefore lower odds of death, especially those with a history of CVD. For example, a study on 10,541 patients hospitalized with COVID-19 at 104 US hospitals showed that treatment with statins prior to admission was associated with a >40% reduction in mortality and a >25% reduction in the risk of developing a severe outcome, after controlling for other medication use, comorbid conditions, hospital site and month of admission, and patient demographic characteristics (35). In another retrospective study of 1,014 patients admitted to the hospital because of COVID-19, 454 patients (44.77%) were treated with statins prior to hospitalization and such therapy was also associated with significant reduction in mortality (36).

However, the evidence regarding the effects of statins in the treatment of COVID-19 are still controversial. A number of clinical trials and observational studies have reported the protective effects of statins in reducing mortality as well as reducing clinical symptoms in respiratory infections (37). Preclinical studies have reported that statins increase ACE2, an enzyme that facilitates the entry of virus into cells. However, this hypothesis has not been proven in humans. Several studies have suggested that statins improve the outcomes of COVID-19 patients by reducing inflammation (38). Anti-inflammatory and immunomodulatory effects of statins are well-known and statins exert their effects by inhibiting the NF-Kβ pathway and by reducing inflammatory mediators such as inflammatory cytokines (IL1, IL6, TNF-α), CRP, and neutrophils. The results of this study indicated that CRP levels were lower in patients treated with atorvastatin than in those who were not treated with statin. It has been shown that atorvastatin, pravastatin, and rosuvastatin can increase ACE2 levels, which supports the hypothesis that statins inhibit RAS activation, improve vascular remodeling after vascular injury and reduce angiotensin II proinflammatory effects. They can alter CD147 expression, structure, and function by inhibiting its isoprenylation and N-glycosylation, and CD147 is a surface protein that can act as a coronavirus receptor. Statins also suppress NLRP3 inflammasome activation and exactly inflammasome activation in SARS-CoV-2 results in respiratory, CV, gastrointestinal, neurological, renal and ophthalmic manifestations as has been very nicely reviewed in a recently published paper by Torres-Peña et al. (39). However, apart from anti-inflammatory and immunomodulatory effects of statins, statins might deplete cholesterol from cell membranes resulting in coronavirus suppression. Namely, a recently published study showed that SARS-CoV-2 induces cholesterol 25-hydroxylase (25HC) both *in vitro* and in COVID-19 infected patients, via interferon signaling and that 25HC activation causes a depletion of accessible cholesterol on cell membrane and results in broad anti-coronavirus activity by blocking viral-cell fusion and preventing viral infection of lung epithelial cells (40).

Neutrophil's count, since they are the main cells involved in inflammation, was lower in patients treated with statin than in those who were not treated with a statin. On the other hand, the lymphocyte count in the statin group was higher. This is in accordance with the results of Zhang et al. who reported that statins reduced CRP levels and neutrophils count (24).

Another study on patients with renal failure who had COVID-19 found that statins reduced neutrophils count, although this had no effect on mortality (41). One of the aims of the present study was to investigate the effects of statins on the hospitalization of patients with COVID-19 in ICU. Results the aim of this study was also to show whether statins had any effect on the rate of hospitalization in ICU. The results of this study could not confirm this. A study by Zhang et al. found that statins reduced admission to ICU. A systematic review and meta-analysis also reported that statins reduced admissions to ICU (14). These discrepancies may be related to the type of statistical methods used and the size of the study population. In this study, most of the participants had comorbidities: hypertension and cardiovascular disease, which was consistent with other studies. Prospective studies are needed to determine the possible benefits of statins in the treatment of COVID-19 patients. Several such clinical trials are ongoing.

## Limitations

Our study as a retrospective study had some limitations. First, because it was retrospective, some medical records of the patients were incomplete. Moreover, the presence of confounding variables caused bias in the results. Therefore, prospective studies should be conducted to obtain more accurate results. Third, this study could only assess the impact of atorvastatin as it was the only statin used by the patients. Hence, it remains unclear if other statins might exert different effects. Finally, the sample size was relatively small to draw a robust conclusion on the impact of statin use on mortality as a single outcome, and larger studies are warranted to explore this further.

## Conclusion

Statins with a wide range of pleiotropic effects besides lipid lowering might be useful in the treatment of COVID-19 patients. The anti-inflammatory effects of statins can contribute to the prevention of cytokine storm and lung damage. The results of this study also suggested the protective effects of statins in reducing inflammation. Statins seem to reduce the composite of mortality, ICU admission, and intubation in COVID-19 patients, although prospective studies are needed in larger populations to confirm this effect.

## Data Availability Statement

The raw data supporting the conclusions of this article will be made available by the authors, upon a reasonable request without undue reservation.

## Ethics Statement

The studies involving human participants were reviewed and approved by the Ethics Committee of Birjand University of Medical Sciences (IR.BUMS.REC.1400.073). The patients/participants provided their written informed consent to participate in this study.

## Author Contributions

HKo, MB, and AS: conceptualization. HKo, HKh, ZK, AN, HM, and HF: data collection and writing-original draft. MA, ŽR, TK, MB, and AS: writing—review and editing. All authors approved the manuscript.

## Funding

This work was supported by Russian Science Foundation (Grant # 22-25-00498).

## Conflict of Interest

MB speakers bureau: Abbott/Mylan, Abbott Vascular, Actavis, Akcea, Amgen, Biofarm, KRKA, MSD, Polpharma, Sanofi-Aventis, Servier and Valeant; consultant to Abbott Vascular, Akcea, Amgen, Daichii Sankyo, Esperion, Freia Pharmaceuticals, Lilly, MSD, Polfarmex, Resverlogix, Sanofi-Aventis; Grants from Sanofi and Valeant. ŽR has received honoraria from Sanofi-Aventis and Novartis. The remaining authors declare that the research was conducted in the absence of any commercial or financial relationships that could be construed as a potential conflict of interest.

## Publisher's Note

All claims expressed in this article are solely those of the authors and do not necessarily represent those of their affiliated organizations, or those of the publisher, the editors and the reviewers. Any product that may be evaluated in this article, or claim that may be made by its manufacturer, is not guaranteed or endorsed by the publisher.
